# Evaluation of parameters related to libido and semen quality in Zebu bulls naturally infected with *Trypanosoma vivax*

**DOI:** 10.1186/s12917-015-0571-x

**Published:** 2015-10-14

**Authors:** Joely FF Bittar, Paula B. Bassi, Dênia M. Moura, Guilherme C. Garcia, Olindo Assis Martins-Filho, André B. Vasconcelos, Matheus F. Costa-Silva, Cristiano P. Barbosa, Márcio SS Araújo, Eustáquio R. Bittar

**Affiliations:** Universidade de Uberaba (UNIUBE), Programa de Mestrado acadêmico em Sanidade e Produçao Animal nos Trópicos, Avenida Nenê Sabino 1697/1698, 38055-500 Uberaba, MG Brasil; Laboratório de Biomarcadores de Diagnóstico e Monitoração, Centro de Pesquisas René Rachou - Fundação Oswaldo Cruz, Avenida Augusto de Lima n° 1715, 30190-002 Barro Preto, Belo Horizonte, MG Brasil

**Keywords:** *Trypanosoma vivax*, Libido, Reproduction, Bull, Semen

## Abstract

**Background:**

Trypanosomiasis is a disease caused by *Trypanosoma (Dutonella) vivax,* a hemoprotozoa that can affect bovines. In South America, the sanguineous form is mechanically transmitted from one mammalian host (ruminant) to another by the bite of a blood-sucking insect or by needles contaminated with infected blood. The negative impact of the parasitosis caused by *T. vivax* infection on the reproductive activity of male and female ruminants is known to reduce fertility. In males, alterations such as degeneration, diffuse or interlobular inflammatory infiltrate found in ovine and bovine testicles, can affect fertility through decreased sperm quality. This study evaluated the impact of natural infection with *T. vivax* on Zebu bulls from the Central Station of Artificial Insemination (CSAI) with regard to libido and the negative effects caused by this protozoan on semen quality.

**Methods:**

Blood samples of 44 animals were collected to evaluate the presence of the trypomastigote form of *T. vivax* in blood smears obtained from hematocrit and buffy coat, and antibody titer IgG anti *T. vivax* in indirect Immunoflorescence (IFI). Furthermore, data related to libido, ejaculate volume, spermatic concentration, and seminal vigor were recorded for these animals employing the criteria of the CSAI.

**Results:**

Nine animals (20.45 %) showed *T. vivax* trypomastigotes and parasitemia between 0.02 and 0.07, and antibody titers from 1:80 to 1:320 in IFI. Twenty nine negative animals in parasitological tests were not reactive in IFI, and six animals presented the antibodies IgG anti *T. vivax* in IFI. Data on reproductive activity showed that animals infected with *T. vivax* have a decreased libido and an increased spermatic volume, whereas other factors related to the reproductive process such as spermatic concentration, motility and spermatic force, were unchanged in infected bulls.

**Conclusions:**

The *T. vivax* infection in Zebu bulls from CSAI caused patent parasitemia, induced a febrile state, promoted reduction in the libido and increased the ejaculate volume. These conditions together may account to decrease the performance of these animals.

## Background

Trypanosomiasis is a disease caused by *Trypanosoma (Dutonella) vivax,* a hemoprotozoan that can affect bovines [1]. In South America, the sanguineous form is mechanically transmitted from one mammalian (ruminant) host to another by the bite of a blood-sucking insect or by fomites, mainly needles contaminated with infected blood. After skin penetration, the protozoon reaches the bloodstream via the lymphatic system and undergoes a prepatent period of 10 to 14 days [2]. *T. vivax* occurs in the majority of tropical countries and represents a potential problem to bovine herds. In Brazil, ruminants naturally infected with *T. vivax* can experience a chronic and asymptomatic infection that is difficult to diagnosis throughout herds. This phenomenon, called trypanotolerance, has a genetic and an environmental component and can vary with age, nutritional status, stress conditions, intercurrent infections, and the strain involved [3, 4]. The disease was introduced in America probably during the 19th century by European colonizers. Studies conducted in Brazil revealed that this hemoparasite has been found in animals in the states of Tocantins [5], Paraíba [6], Maranhão [7], Minas Gerais [8], and mainly in the region of the Pantanal in Mato Grosso [9].

The negative impact of *T. vivax* on the reproductive activity of male and female ruminants has been shown to result in a reduction in fertility with a significant impact on herd productivity [2, 10]. The clinical signs of trypanosomiasis include anemia, fever, lethargy, progressive weight loss, decrease in fertility, reduced production of milk and meat, abortions, agalaxia, and eventually death [11, 12].

Trypanosomiasis is responsible for many reproductive disorders in bovine herds such as the degeneration of the hypothalamus, the pituitary gland, and the gonads causing, as a consequence, alterations to the concentration of secretions and plasmatic concentrations of essential hormones for reproduction in both males and females [2, 10]. In males, alterations such as degeneration, diffuse or interlobular inflammatory infiltrate found in ovine and bovine testicles, can directly affect the fertility of the animals through decreased sperm quality. The affected parameters are volume, concentration, viability, motility and spermatic pathology, showing that not only females infected with *T. vivax* have reproductive issues [13, 14].

As there is little information regarding the effect of *T. vivax* on the libido or semen volume and quality of Zebu bulls in Brazil, this study aimed to diagnose the presence of *Trypanosoma vivax* infection from donator bulls from the Central Station of Artificial Insemination (CSAI) and verify the negative effects caused by this protozoan on libido and semen features in naturally infected bulls.

## Methods

### Animals

This work was conducted at the Veterinary Hospital of Uberaba and in a laboratory associated with the Central Station of Artificial Insemination (CSAI) in Uberaba, Minas Gerais, Brazil. Forty-four Zebu bulls (*Bos indicus*) in reproductive age varying from 3 to 14 years from the CSAI were evaluated. The presence of the trypomastigote form of *T. vivax* had already been detected at CSAI from blood smears of peripheral blood of a cow used in a practical artificial insemination class. The bulls received a daily diet of 30 k of ground forage grass (Napier grass) and 4 k of maintenance feed in addition to Estrela grass, which was the natural paddock hay, and water *ad libitum.* At the time of blood collection, rectal temperature was measured and a clinical examination of the animals was performed (behavior, palatability and mucosa color). This study protocol was approved by the Animal Experimentation Ethical Committee from Universidade de Uberaba, Uberaba, Minas Gerais, Brazil (CEEA/UNIUBE, protocol number 001/2013) and followed all the ethical principles of cattle experimentation procedures.

### Blood sampling and assessment of parasitemia

For the identification of the blood trypomastigote form of *T. vivax,* blood samples were collected by jugular venipuncture into sterile vacuum tubes containing an anticoagulant (EDTA 7.2 mg). Blood was also collected into sterile vacuum tubes without anticoagulant for further investigation of the antibodies IgG anti-*T. vivax* using the technique of indirect immunofluorescence, adapted from the classical method proposed by Camargo et al. [15].

To identify *T. vivax*-positive animals, observations were performed using the methodology described by Woo [16]. For each animal, two micro-hematocrit tubes were filled to 2/3 of their volume and had the rear end sealed by flame. The tubes were then centrifuged at 15,000 *g* to obtain the buffy coat suspension. Blood smears were made by breaking the micro-hematocrit tubes in the area of transition between plasma and leukocyte and placing the part containing the erythrocytes on the slide for the smear confection. The blood smear was dyed with quick panoptic (Hematocor-Biocor®). Slides were subsequently observed using an optical microscope with a 100x immersion oil objective. The calculation of parasitemia of positive animals was performed following the method described by Brener [17], which involved determining the number of *T. vivax* observed in ten fields of view, multiplying this number by 100 and dividing it by the number of red blood cells in all ten fields (+/−1000).

Trypomastigotes observed in blood smears were measured using the morphometric program (Image Tool®). Measurements and observations recorded were: total length of parasite including free flagellum (L), distance from the end of posterior extremity to kinetoplast (PK), distance from kinetoplast to the middle of the nucleus (KN), distance from the end of posterior extremity to the middle of the nucleus (PN), distance from the middle of the nucleus to the end of anterior extremity (NA), length of free flagellum (F), kinetoplast index (KI), and nuclear index (NI). Measurements were made following the method described by Hoare [18] and the trypomastigotes were compared with those described from Brazil by Carvalho [8], Linhares [5], Shaw & Lainson [19], Silva et al. [9] and Paiva et al. [20], in the Table 1.

**Table 1:** Comparison of biometric averages of *Trypanosoma vivax* from Zebu bulls from the Central Station of Artificial Insemination (CSAI) and other isolates in Brazil^a^

Locality	L	PK	KN	PN	NA	F	KI	NI
Minas Gerais (CSAI) - Present study	20.56	0.86	5.44	6.31	5.62	6.44	1.16	1.54
Minas Gerais [8]	19.89	0.90	6.64	7.55	7.46	6.22	1.04	1.14
Tocantins [5]	19.42	0.96	6.24	7.25	5.87	6.29	1.16	1.24
Pará [18]	22.77	0.65	6.16	7.60	8.22	6.92	1.23	0.94
Mato Grosso [9]	18.73	1.02	6.10	7.18	5.40	6.15	1.17	1.50
Mato Grosso do Sul [19]	18.10	0.30	7.46	7.76	6.03	4.30	1.04	1.34

^a^Data are presented in μm. *L* total length of parasite, including free flagellum, *PK* distance from the end of posterior to kinetoplast, *KN* distance from kinetoplast to the middle of the nucleus. *PN* distance from the end of posterior extremity to the middle of the nucleus, *NA* distance from the middle of the nucleus to the end of anterior extremity. *F* free flagellum length, *KI* kinetoplast index, *NI* nuclear index

### Serological test (indirect immunofluorescence - IFI)

To perform indirect immunofluorescence, slides containing trypomastigotes of *T. vivax* were used. The slides were produced using ovine blood that was experimentally infected. The experimental infection followed the ethical principles of animal experimentation adopted by Ethics in Animal Experimentation Committee (CEEA/UNIUBE, protocol number 035/2012). At the highest parasitemia (2 × 10^7^ trypanosomes/mL of blood) 100 mL of blood was collected in vacuum tubes containing EDTA. Parasites were obtained following the protocol described by Cuglovici et al. [21], with modifications. The blood was mixed with Percoll Solution (Sigma®) in same proportion and centrifuged at 17,500 *g* for 20 min at 4 °C. The parasite layer formed near the top of the gradient and was collected and re-suspended in phosphate buffered saline (PBS of NaH2PO4 40 mM, pH 7.5 and NaCl 150 mM) in the proportion of 1:3. The suspension was then centrifuged at 6000 *g* for 15 min at 4 °C, the supernatant discarded, and the sediment washed twice with PBS 6000 *g* for 15 min at 4 °C aiming to remove any remaining of Percoll. The partially purified trypanosomes were distributed on pre-demarcated glass slides. After natural drying, the slides were fixed in cold acetone for 5 min and again naturally dried, wrapped in paper tissues and aluminum foil, and stored at −20 °C until the moment of use.

The serums obtained from animals to be tested were diluted in the ratio 1:2 to1:640 in PBS. The detection of the antibodies IgG anti-*T. vivax* was done employing a rabbit antibody anti-bovine IgG conjugated with fluorescein isothiocyanate (Sigma St Louis, MO, USA) in dilution of 1:200 in PBS. Serum samples that were reactive in dilution 1:80 or greater were considered positive [20].

### Hematological analysis

The analysis of Red Blood Cell (RBC) included erythrocyte counts, hemoglobin concentration, hematocrit, Mean Corpuscular Volume (MCV), Mean Corpuscular Hemoglobin Concentration (MCHC). The White Blood Cell (WBC) included total and differential leukocyte counts. All hematological measurements were carried out in a hematology analyzer (ABC VET - Horiba® ABX Diagnostics). The differential leukocyte count was performed by optical microscopy (Nikon Eclipse E200®), 1000× magnification, of blood smears stained with Fast Panoptic kit (Laborclin®, São José do Rio Preto - SP).

### Evaluation of features related to reproductive activity

The data concerning libido, ejaculate volume, seminal concentration, and spermatic vigor were obtained following the CSAI criteria. For evaluation of libido, the methodology described by Fonseca & Chow [22], was followed with some modifications. Five cows were brought to estrus after application of prostaglandin (PGF_2_α). The bulls were individually tested for a 3-h time frame and their performance was documented every 5 min. Libido was classified using the table of libido classification for Zebu bulls of the Brazilian College of Animal Reproduction [23]: 0 to 3 = questionable; 4 to 6 = good; 7 to 8 = very good; 9 to 10 = excellent or superior.

The semen was analyzed for volume, concentration, motility and spermatic vigor the standards set by the Ministry of Agriculture and the Brazilian College of Animal Reproduction [23]. Sperm collection employed an artificial vagina and the evaluation was done individually.

### Statistical analysis

Data were reported as means and standard deviations and analyzed using the student *t* test with graph pad prism® version 5.0 software (GraphPad Software, San Diego, CA, USA). Data were considered statistically different with *P* < 0.05.

## Results

**Fig. 1:**
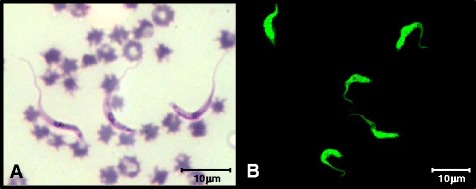
**a** Trypomastigotes of *T. vivax* in a blood smear of an infected animal dyed with Quick Panoptic and seen using an optical microscope 1,000× magnification. **b** The form of trypomastigotes marked with fluorescein isothiocyanate from indirect immunofluorescence of a positive animal. Image obtained using confocal microscope (Zeiss® LSM 510 Meta) 1000× magnification

Evaluation of the blood smears allowed the identification of the trypomastigote form of *T. vivax* (Fig. 1a) in 9 of 44 animals (20.5 %) with parasitemia ranging from 0.02 to 0.07 %. The biometric data of the trypomastigotes found in positive samples were compared to the averages found in positive samples from other parts of Brazil and are described in Table 1.

The indirect immunofluorescence test found 15 animals (34.1 %) with the antibody titers IgG anti-*T.vivax* varying from 80 to 320 (Fig. 1b); 29 of 44 animals (65.9 %) were negative. The nine animals that were found positive through blood smears were also found positive by indirect immunofluorescence. Six animals that were found negative through blood smears presented the antibodies IgG anti-*T. vivax* in the indirect immunofluorescence test, with antibody titers varying from 80 to 160. These six animals were excluded from further analysis.

Hematological analysis demonstrated that in general no significant differences were observed in the erithrogram and leukogram in both groups of animals. A discreet lymphocytosis, as compared to the reference values [24] were observed in both groups of animals (Table 2).

**Table 2:** Hematological records in *T. vivax*-infected Zebu bulls

Parameters	Non-infected	*T. vivax*-infected
Red Blood Cells (RBC)		
Erythrocytes (cells/mm^3^) (5-10 × 10^6^)	8.2 ± 1.1 × 10^6^	7.8 ± 1.1 × 10^6^
Hemoglobin (g/dL) (8–15)	11.7 ± 1.9	11.3 ± 1.9
Hematocrit (%) (24–46)	34.5 ± 6.4	34.0 ± 6.5
MCV (fL) (40–60)	42.6 ± 8.6	35.7 ± 17.7
MCHC (g/dL) (30–36)	33.3 ± 2.0	33.4 ± 1.3
White Blood Cells (WBC)		
Total leukocytes (cells/mm^3^) (4 − 12 × 10^3^)	11.6 ± 2.4 × 10^3^	11.8 ± 4.1 × 10^3^
Eosinophils (cells/mm^3^) (0 − 2.4 × 10^3^)	0.27 ± 0.16 × 10^3^	0.45 ± 0.15 × 10^3^
Neutrophils (cells/mm^3^) (0.6 − 4 × 10^3^)	2.2 ± 1.0 × 10^3^	2.2 ± 1.4 × 10^3^
Monocytes (cells/mm^3^) (0.03 − 0.84 × 10^3^)	0.24 ± 0.18 × 10^3^	0.27 ± 0.19 × 10^3^
Lymphocytes (cells/mm^3^) (2.5 − 7.5 × 10^3^)	8.5 ± 1.7 × 10^3a^	9.1 ± 3.9 × 10^3a^

Data presented as mean ± standard deviation; Reference Values [24] are provided below each parameter; ^a^indicate values outside the reference values

**Fig. 2:**
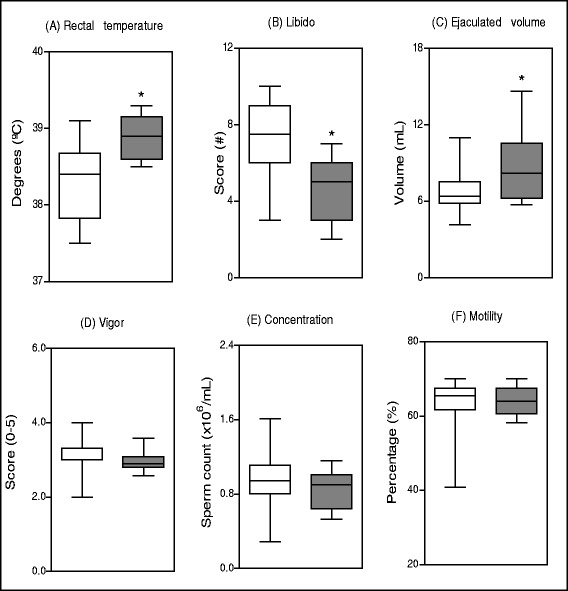
Mean Values of rectal temperature (**a**), libido (**b**), ejaculate volume (**c**), spermatic vigor (**d**), spermatic concentration (**e**) and spermatic motility (**f**) in ejaculates of bulls non-infected (white rectangle symbol) and naturally infected with *T. vivax* (grey rectangle symbol). *Significant differences at *p* < 0.05 between non-infected and infected groups. # According to table of libido classification for Zebu bulls of the Brazilian College of Animal Reproduction [23]: 0 to 3 = questionable; 4 to 6 = good; 7 to 8 = very good; 9 to 10 = excellent or superior

Our data demonstrated that out of the 38 animals analyzed for clinical and reproductive parameters, 76.3 % (29/38) were considered not infected and 23.7 % (9/38) were classified as infected. The mucosa coloration, behavior, and palatability did not show any considerable variation in positive bulls when compared to negative bulls. However, rectal temperature was greater in infected animals when compared to non-infected animals, 38.9 ± 0.3 °C and 38.4 ± 0.5 °C (*p* < 0.05), respectively (Fig. 2a). Libido evaluation found that infected bulls had higher score, ranging from 2.0 to 7.0 (5.0 ± 1.6), than the non-infected bulls, ranging from 3.0 to 10.0 (7.5 ± 2.1) (Fig. 2b). According to the table of libido classification, the Zebu bulls of this study revealed that infected bulls had libido values that were considered “questionable” to “good” whereas the non-infected animals had libido values that were considered “very good” and “excellent”. Statistically, the libido of positive bulls was inferior (*p* < 0.05) to that of negative bulls. The results for ejaculate volume showed that infected bulls had higher collected volume when compared to non-infected animals, 8.2 ± 2.7 mL and 6.4 ± 1.8 mL (*p* < 0.05), respectively (Fig. 2c).

The evaluation of sperm vigor did not show any difference between infected and non-infected animals with score averages ranging from of 2.6 to 3.2 (2.9 ± 0.3) and 2.0 to 4.0 (3.0 ± 0.4), respectively (Fig. 2d). Spermatic concentration of infected and non-infected animals also did not differ significantly between infected and non-infected animals, ranging from 0.5 to 1.2 (0.9 ± 0.2 sperm counts × 10^6^/mL) and 0.5 to 1.6 (0.9 ± 0.3 sperm counts × 10^6^/mL), respectively (Fig. 2e). Spermatic motility varied from 58.2 to 70.0 % (64 ± 3.8) for infected animals, and from 33.3 to 70.0 % (65.3 ± 8.5) for non-infected animals, which were not significantly different (Fig. 2f).

## Discussion

There are many techniques for diagnosing infection with *T. vivax* such as blood smears, lymph node aspiration, and inoculation of mice, however, we found that the micro-hematocrit technique described by Woo [16] to be effective at discriminating infected animals. The biometric results for the parasites in this study were similar to that found in other *T. vivax* isolates from other Brazilian states [8] thus confirming the morphometric diagnosis of *T. vivax* in bulls from CSAI [18].

The seroprevalence of 34.1 % for the sample of bulls from CSAI was significant. However, the prevalence at CSAI was lower than what was found by Madruga et al. [25], who verified a prevalence of 37.7 % in cities from the state of Pará and 56.0 % in the state of Mato Grosso do Sul, and Cuglovici et al. [20], who found a prevalence of 35.7 % in the region of Igarapé, state of Minas Gerais.

The infected animals in this study did not show clinical symptoms of trypanosomiasis, which can be explained by the good physical and nutritional status of the animals. A similar observation was made by Schenk et al. [26], who indicated that good nutritional status establishes equilibrium between parasite and host (trypanotolerance), and therefore the animal is clinically normal. In addition, Batista et al. [4] and Osório et al. [27] reported a natural resistance to the infection in bovines from Latin America.

In this study, we did not find any hematological alterations associated with the *T. vivax* infection. A discreet lymphocytosis, as compared to the reference values was observed in both groups of animals which may be a consequence of animal management [28] at the Central Station of Artificial Insemination (CSAI).

According to studies developed by Almeida et al. [29], Batista et al. [30] and Schenk et al. [26], animals with the highest level of parasitemia experience an increase in body temperature, however, the significant increase in rectal temperature of positive animals in the present study was within the interval considered as “normal” as described by DuPreez [31], which can, again, be justified by good nutrition according to observations of Van Den Bossche & Rowlands in 2001 [3].

The results of the present study showed that *T. vivax* produces a negative effect in the libido of infected animals. According to Schenk et al. [26], this parasitism can cause a decrease in the libido especially during febrile periods and in condition of hypoxia. The average rectal temperature in positive bulls from the CSAI was higher than that observed in non-infected bulls. We hypothesized the febrile state triggered by the *T. vivax* infection would contribute to an impaired general clinical status of Zebu bulls and subsequently compromised their reproductive performance. This hypothesis was constructed based on previous findings reported by Schenk et al. [26], Setchell [32] and Bezerra & Batista [11]. The latter have demonstrated that T. vivax infection can lead to reproductive pathologies in male leading to decreased semen quality. In chronic cases of disease, infertility or even sterility might be present [11]. According to Setchell [32] hyperthermia, anorexia and anemia caused by *T. vivax* contribute to the triggering of the degeneration of reproductive organs. The conditions observed in the infected animals in our study, could have led to testicular degeneration and, consequently, compromised the performance of animals in semen production. Ultimately, it is possible that these conditions of infection could have negative impact on the reproductive capacity of the animals. Bezerra & Batista [11] described that bovines with increased body temperature for a long period of time experienced a reduction in plasmatic concentration of testosterone. This fact could be a consequence of many protozoan produced events in the testicles that would explain the reduction of libido in the positive animals.

According to Galloway [33], ejaculated volume can vary between 2 to 6 mL due to the method of sample collection when using an artificial vagina in Zebu. However, variation sometimes also depends on the animal itself such as deficiency in the contraction of deferent vessels and the epididymis tail in response to stimulus. Sekoni et al. [14] and Adamu et al. [13] have reported that *T. vivax*-infected presented reduced ejaculated volume. Adamu et al. [13] reported damage to the accessory gland of males associated with the toxins released locally by the parasite, such as hemolysins, inflammatory and permeability factors, factors activating the complement system, immunosuppression factors, and substances liberated by dead trypanosomes. In this study, we have found that the average ejaculated volume was higher in *T. vivax* positive bulls and associated with a reduction of spermatic concentration. These findings may be related to *T. vivax*-related pathological conditions previously reported by Bezerra e Batista [11] such as interstitial perivascular epididimitis or with hyperplasia of epididimal epithelium cause by the parasite as reported by Adamu et al. [13]. These pathological conditions are associated with increased body temperature [32], as observed in the *T. vivax*-infected bulls included in our investigation.

## Conclusion

Our results demonstrated that the *T. vivax* infection in Zebu bulls from CSAI caused patent parasitemia, induced a febrile state, promoted reduction in the libido and increased the ejaculate volume. These conditions together may account to decrease the performance of these animals.

## Abbreviations

CEEA: Ethics in Animal Experimentation Committee
CSAI: Central Station of Artificial Insemination
EDTA: Ethylenediamine tetraacetic acid
F: Length of free flagellum
FITC: Fluorescein isothiocyanate
*g*: Gravity (centrifugal force)
IFI: Indirect Immunoflorescence
IgG: Immunoglobulin G
KI: Kinetoplast index
KN: Kinetoplast distance from kinetoplast to the middle of the nucleus
L: Total length of parasite including free flagellum
MCHC: Mean Corpuscular Hemoglobin Concentration
MCV: Mean Corpuscular Volume
NA: Distance from the middle of the nucleus to the end of anterior extremity
NI: Nuclear index
PBS: Phosphate buffered saline
PK: Distance from the end of posterior extremity to kinetoplast
PN: Distance from the end of posterior extremity to the middle of the nucleus
RBC: Red Blood Cell
WBC: White Blood Cell
